# Infant temperament, maternal feeding behaviours and the timing of solid food introduction

**DOI:** 10.1111/mcn.12771

**Published:** 2019-01-29

**Authors:** Samantha L. Rogers, Jackie Blissett

**Affiliations:** ^1^ Centre for Research in Public Health and Community Care University of Hertfordshire Hatfield U.K.; ^2^ Department of Psychology Aston University Birmingham U.K.

**Keywords:** complementary feeding, infant feeding, infant feeding behaviour, infant temperament, postnatal depression, solid food introduction

## Abstract

Despite guidance from the World Health Organization and the U.K. Department of Health, many mothers introduce solid food before their infant is 6 months old. The current study aimed to investigate relationships between maternal feeding behaviours (preintroduction and postintroduction to solids), infant temperament, and the timing of introduction to solid food. Eighty‐one women were recruited on low‐risk maternity units and were contacted at 1 week, 3, and 6 months postpartum. Mothers of infants (45 males, 36 females, mean birth weight 3.52 kg [*SD* 0.39]) completed the behaviours component of the Infant Feeding Style Questionnaire via telephone interview at 3 months. At 6 months, they were observed feeding their infant solid food at home and reported infant temperament using the Infant Behaviour Questionnaire‐Revised (short form). Partial correlations (covariates: birth weight, maternal age, breastfeeding duration, and postnatal depression) revealed negative associations between age of introduction to solid food and temperament (smiling and laughter) and laissez‐faire milk feeding behaviours; and positive associations between age of introduction to solid food and restrictive milk feeding behaviours and verbal involvement during an observed mealtime. Hierarchical multiple regression analysis revealed that an infant's birth weight and the degree to which their mothers perceive them to smile and laugh are key predictors of when they will be introduced to solid food, over and above other variables of interest (e.g., maternal milk feeding behaviours, breastfeeding duration, and postnatal depression).

**Key messages**

- Infants introduced to solid food earlier were heavier at birth, had younger mothers who breastfed for shorter durations (or not at all), and scored lower for postnatal depression.
- Mothers who introduced solid food earlier reported not keeping track of how much milk their infant drank and using less restrictive feeding behaviours at 3 months.
- Mothers who introduced solid food earlier were observed to initiate less (or no) conversation or spontaneous comments during a mealtime at 6 months.
- Earlier solid food introduction was best predicted by heavier birth weight and higher ratings for smiling and laughter (during play or general caretaking).

## 1 BACKGROUND

Paediatric obesity is one of the major international public health challenges this century. In 2015, 28% of 2‐ to 15‐year‐olds in England were classed as overweight or obese (Conolly, 2016). Research has found that shorter durations of breastfeeding and earlier introduction to solid food are associated with faster weight gain and heavier weight in infancy and childhood (Arenz, Rückerl, Koletzko, & Von Kries, 2004; Baker, Michaelsen, Rasmussen, & Sorensen, 2003; Hornell, Lagstrom, Lande, & Thorsdottir, 2013; McCrory & Layte, 2012; Owen, Martin, Whincup, Smith, & Cook, 2005; Rogers & Blissett, 2017). However, findings are mixed, and some studies have failed to find these effects (Moorcroft, Marshall, & McCormick, 2011; Novaes, Lamounier, Colosimo, Franceschini, & Priore, 2012). Research investigating factors associated with longer breastfeeding and later introduction to solid food are therefore essential and will enable the development of more effective interventions and prevention programmes.

The World Health Organization (WHO) and U.K. Department of Health (DH) recommend infants be exclusively breastfed until 6 months old, at which point complementary foods should be introduced (DH, 2003; WHO, 2002). Despite this guidance, the U.K. has the poorest breastfeeding rates in the world (Lancet, 2016). The most recent Infant Feeding Survey illustrated that although 81% of women initiated breastfeeding after giving birth, rates of any breastfeeding fell quickly to 69% at 1 week, 55% at 6 weeks, and 34% at 6 months (McAndrew et al., 2010). Furthermore, only 1% of mothers were exclusively breastfeeding at 6 months (McAndrew et al., 2010). In addition, 30% of mothers had introduced solid food by 4 months and 75% by 5 months (McAndrew et al., 2010).

Research has shown that feeding attitudes and decisions are affected by a range of factors. A recent meta‐analysis revealed that factors consistently found to influence breastfeeding decisions include maternal smoking, educational attainment, and whether they remained close to their infant during their hospital stay (Cohen et al., 2018). Mothers with more favourable attitudes towards breastfeeding also tend to report higher household incomes and be employed and married to (or cohabiting with) their partner (Sittlington, Stewart‐Knox, Wright, Bradbury, & Scott, 2007). Guidance from the DH states a baby is ready for solid food if they can (a) stay sitting and hold their head steady; (b) look at food, pick it up, and put it in their mouth by themselves; and (c) swallow food, rather than push it out of their mouth (NHS, 2011). Despite this guidance, Brown and Rowan (2016) found that mothers' reasons for introducing solid food included perceptions that infants were hungry, unsettled or not getting enough sleep. Research has found that increasing the calories infants consume during the day, by either consuming more milk or solid food, does not reduce the number of times infants wake during the night (Brown & Harries, 2015). The current literature lacks studies that investigate other predictors of timing of introduction to solid food, such as maternal perception of infant individual differences.

One factor, which may be important in infant feeding, is that of perceived temperament. Infant temperament is related to weight gain and feeding outcomes during infancy and the preschool years; more rapid weight gain is associated with difficulty (more intense, lower mood, slower to adapt to new situations, difficult to soothe), distress (crying/fussing), surgency/extraversion (preference for stimulation, sensation seeking), and emotionality (easily distressed, inhibited; Bergmeier, Skouteris, Horwood, Hooley, & Richardson, 2014). Furthermore, infant difficulty significantly predicts feeding difficulties at 6 months (Farrow & Blissett, 2006), and children who display greater emotionality and shyness tend to be more neophobic and exhibit more food avoidant behaviours (Blissett & Fogel, 2013; Haycraft, Farrow, Meyer, Powell, & Blissett, 2011; Pliner & Loewen, 1997). There is also a small emerging literature that has explored infant temperament in relation to the timing of introduction to solid food. Tatone‐Tokuda, Dubois, and Girard (2009) found that maternal perception of difficult infant temperament did not significantly predict early complementary feeding; yet, Wasser et al. (2011) found that infants were more likely to be introduced to solid food before 4 months if their mothers rated them as higher in distress and activity.

Feeding decisions, including the timing of introduction to solid food, are also associated with parental feeding styles. For example, mothers who breastfeed their infants for longer durations report greater responsiveness to their infant's cues and use less controlling feeding styles when feeding solid food (DiSantis, Hodges, & Fisher, 2013). Mothers who breastfeed for longer also display more sensitivity towards their infant during observed mealtimes (DiSantis et al., 2013; Rogers & Blissett, 2017). These feeding styles are driven by a range of factors, such as anxiety. For example, mothers who cease breastfeeding commonly report worrying over not knowing the amount of milk that is consumed during a feed and not being able to predict feeding times (Brown, 2018). Interestingly, there has been only one study that has examined the contribution of both infant temperament and maternal feeding style to timing of introduction to solid food (Doub, Moding, & Stifter, 2015).

Doub et al. (2015) found that infants introduced to solid food earlier had mothers who were younger, were less educated, were heavier (prepregnancy), breastfed for shorter durations, reported lower responsiveness to their infants' hunger and satiety cues, and reported believing infants <6 months need more than milk. Infant temperament moderated the effect of maternal feeding style on solid food introduction; mothers of highly active infants, who believed young infants need more than milk, introduced solids earlier than mothers who did not endorse this feeding style. Doub et al. (2015), however, only assessed feeding styles after solid foods had been introduced, and mother–infant dyads were grouped by method of milk feeding (breastfed, mixed fed, and formula fed) at 4 months postpartum. Grouping mixed fed infants together means that it is not possible to distinguish between infants who receive predominantly breast milk and infants who receive predominantly formula. Alternatively, measuring the duration of any breastfeeding, as a continuous variable, may be advantageous given that previous studies have shown a longer duration of (any) breastfeeding is associated with slower weight gain and a reduced risk of paediatric obesity (McCrory & Layte, 2012; Rogers & Blissett, 2017). In addition, Doub et al. (2015) did not incorporate a naturalistic mealtime observation to validate parental report of feeding styles; this is important, as some parents may be unaware of the feeding styles they use with their infants (Bergmeier, Skouteris, Haycraft, Haines, & Hooley, 2015).

Furthermore, other factors may confound the relationship between infant feeding and temperament, such as postnatal depression. Previous research has shown that postnatal depression is associated with more negative attitudes towards breastfeeding, shorter durations of exclusive breastfeeding, and earlier introduction to solid food (Abou Nazel & Nosseir, 1994; Hampson, Tonstad, Irgens, Meltzer, & Vollrath, 2009). Research has also demonstrated that mothers with postnatal depression report having more difficult infants at 2 and 6 months postpartum (McGrath, Records, & Rice, 2008). This would suggest that research examining the potential predictors of timing of introduction to solid food needs to include a wider range of maternal and infant factors.

The aims of this study were to (a) investigate the relationships between maternal feeding behaviours (during both exclusive milk feeding and after the introduction of solid food), infant temperament, and the age of introduction to solid food and (b) establish the degree to which these variables predict the age of introduction to solid food in addition to covariates, such as infant birth weight, postnatal depression, and maternal age. Based on previous research that has examined infant temperament, eating and weight, it was hypothesised that infants perceived to be more physically active, who show more distress and sadness and who are more difficult to soothe would be introduced to solid food earlier. It was also hypothesised that mothers who reported using milk feeding behaviours characterised by more pressure and less responsiveness and who were observed to be more controlling when feeding solid food, would introduce solid food earlier.

## 2 METHODS

The study protocol received full ethical approval from Birmingham East, North, and Solihull Research Ethics Committee, United Kingdom (reference number 10/H1206/67). Research and Development approval was granted by Birmingham Women's National Health Service Foundation Trust (reference number 10/BWH/NO95).

As part of a wider longitudinal study of infant feeding (Rogers & Blissett, 2017), 287 women were approached after delivery on low‐risk maternity units of Birmingham Women's Hospital. Of these, 81 mothers, mean age 29.42 years (standard deviation [*SD*] 5.87), gave informed consent and agreed to be visited at home. Infants born prematurely (prior to 36‐week gestation) or small for gestational age were excluded. Mothers were required to read and write English. Data presented in this paper were collected at 1 week (demographics only), 3 (feeding questionnaire), and 6 months (questionnaires and mealtime observation) postpartum.

Mothers of infants (45 males, 36 females; mean birth weight 3.52 kg [*SD* 0.39]) reported demographics at 1 week. Feeding information (exclusivity and duration of breastfeeding; when solids were first introduced) and symptoms of postnatal depression were reported at 1 week, 3, and 6 months. Mothers self‐reported feeding behaviours via telephone interview at 3 months, were observed feeding their infant solid food at home at 6 months and reported infant temperament at 6 months. Mothers and infants were weighed and measured at 1 week and 6 months.

### 2.1 Demographic information

Mothers reported their age, prepregnancy weight, ethnic background, household income, educational level, and infant date of birth. Mothers also reported smoking status and whether they had initially planned to breastfeed, formula feed, or mix feed their baby.

### 2.2 Feeding information

At each visit, mothers reported whether infants were being breast fed or formula fed and the duration and exclusivity of feeding method. At 3 and 6 months, mothers reported whether/when they had introduced solid foods.

### 2.3 Infant Feeding Style Questionnaire

The Infant Feeding Style Questionnaire (IFSQ; Thompson et al., 2009) is a validated parental report measure of feeding style during infancy (Thompson et al., 2009). The behaviour component of the IFSQ was administered via telephone interview at 3 months to assess maternal feeding behaviours during the period of exclusive milk feeding. The behaviour component of the IFSQ was modified so that only questions deemed appropriate for the period of exclusive milk feeding were asked. Feeding behaviours assessed include laissez‐faire (parent does not limit diet quality or quantity, little interaction with infant during feeding), pressuring (parent is concerned with increasing amount of milk infant consumes and feeds to soothe infant), restrictive (parent limits quantity of milk consumed), and responsive (parent monitors diet quality and attends to infant's hunger and satiety cues). Mothers reported their feeding behaviours using a five‐point scale, ranging from 1 (*Never*) to 5 (*Always*) or (*Not Applicable*). The Cronbach's α values were 0.72 for pressuring, 0.73 for restrictive, and 0.56 for responsive.

### 2.4 Infant Behaviour Questionnaire‐Revised (short form)

Mothers completed the Infant Behaviour Questionnaire‐Revised (IBQ‐R; Putnam, Helbig, Gartstein, Rothbart, & Leerkes, 2014) at 6 months. The IBQ‐R is a caregiver report measure of temperament and is suitable for 3‐ to 12‐month‐old infants. It consists of 91 items, which comprise 14 subscales. Each item has eight responses to choose from 1 (*Never*) to 7 (*Always*) and X (*Does not apply*) and describes infant behaviour over the previous 7 days. Table 1 provides a description of each subscale along with its Cronbach's α value.

**Table 1:** Subscale descriptions of the IBQ‐R (short form; Putnam et al., 2014)

IBQ‐R subscale	Description	Cronbach's α
Activity level	Motor activity, including movement of arms and legs, wriggling, and squirming	0.71
Distress to limitations	Crying, fussing, or distress whilst confined, being cared for, or when unable to complete a desired action	0.77
Fear	Startle or distress to sudden changes in stimulation, new objects, or social stimuli.	0.81
Duration of orienting	Attention to (or interaction with) a single object for prolonged periods of time	0.78
Smiling & laughter	Smiling or laughter during play or caretaking activities	0.86
High intensity pleasure	Pleasure or enjoyment related to high stimulus intensity, complexity, novelty, and incongruity	0.78
Low intensity pleasure	Pleasure or enjoyment related to low stimulus intensity, complexity, novelty, and incongruity	0.62
Soothability	Decline in crying, fussing, or distress when soothed by the caregiver	0.68
Falling reactivity	Amount of recovery from peak distress, excitement, or general arousal; easiness of falling asleep	0.85
Cuddliness	Expressed enjoyment and moulding of the body when held by a caregiver	0.77
Perceptual sensitivity	Frequency that slight, low intensity, stimuli from the external environment is detected	0.89
Sadness	General low mood; reduction in mood and activity related to personal suffering, physical state, object loss, or when unable to perform a desired action	0.71
Approach	Excitement and rapid approach to pleasurable activities.	0.83
Vocal reactivity	Vocalisations displayed during daily activities	0.65

*Note*. IBQ‐R: Infant Behaviour Questionnaire‐Revised.

### 2.5 Edinburgh Postnatal Depression Scale

Postnatal depression was assessed as a potential covariate (and treated as a continuous variable) in the current study. The Edinburgh Postnatal Depression Scale (EPDS; Cox, Holden, & Sagovsky, 1987 was given to mothers at 6 months. It consists of 10 short statements, each of which has four responses to choose from, indicating how the mother has felt during the previous week. Mothers who score 10 or greater are identified as showing symptoms indicative of possible depression. The EPDS had good internal consistency (Cronbach's α 0.83).

### 2.6 Feeding Interaction Scale

The Feeding Interaction Scale (FIS; Wolke, Sumner, McDermott, & Skuse, 1992) was used to code maternal behaviours during the mealtime observation (Table 2 details subscales used and behaviours assessed). This FIS has clinical validity and has been used to assess maternal–infant feeding interactions and diagnose feeding problems (Farrow & Blissett, 2005; Lindberg, Bohlin, Hagekull, & Palmerus, 1996; Rogers & Blissett, 2017; Rogers, Ramsay, & Blissett, 2018; Skuse, Wolke, & Reilly, 1992).

**Table 2:** Subscales used from the FIS (Wolke et al., 1992)

Subscale	Behaviour	Scoring	Intraclass correlation coefficients
Maternal verbal involvement	Proportion of session mother is talking to infant including initiating conversation and spontaneous comments	1 (*never talks to infant*) to 9 (*very much*)	0.96
Verbal control behaviour	Extent that mother interacts in a controlling manner. For example, verbalisations channel infant behaviour in specific directions	1 (*very high*) to 9 (*very low*)	0.98
Nonverbal maternal behaviour	Extent that mother tries to control outcome of mealtime. For example, forcing or distracting infant to eat	1 (*50% or more time force feeding*) to 9 (*75% of time supervising child actively feeding themselves*)	0.82
Appropriateness of maternal mealtime behaviour	Feeding is appropriate if it is pleasurable for mother and infant.	1 (*very inappropriate*) to 5 (*very appropriate*)	0.84
Maternal sensitivity	Infant in sensible position including freedom of arm movement and eye contact with mother, close proximity to mother, feedback on infant's behaviour, variation of stimulation	1 (*highly insensitive*) to 9 (*highly sensitive*)	0.77

*Note*. FIS: Feeding Interaction Scale.

Mealtime observations took place in participants' homes and were recorded using a video camcorder and tripod. Mothers decided what food to feed their infant and were advised to feed their infant as normal. Infants tended to be fed foods that they had eaten at least a few times before (84.5%) and that mothers perceived them to like (82%). Videos were independently scored later by the researcher and a research assistant. Each recording was viewed, and maternal behaviour was rated, in 2‐min interval sections. A mean for each FIS subscale was calculated once the full recording had been viewed. Thirty‐three percent of videos were double scored; intraclass correlation coefficients can be found in Table 2.

### 2.7 Data analysis

Statistical analyses were undertaken using IBM SPSS v24. Pearson's correlations were used to assess which demographic variables were related to age of introduction to solid food. Partial correlations were then used to investigate the possible relationships between variables whilst controlling for significant covariates. One‐tailed partial correlations were used to assess the relationship between (a) age of introduction to solid food and infant temperament, (b) age of introduction to solid food and maternal report of feeding behaviours during exclusive milk feeding at 3 months, and (c) age of introduction to solid food and observed maternal mealtime behaviours during solid food feeding at 6 months. Finally, hierarchical multiple regression analysis was performed to predict age of introduction to solid food using predictors significantly correlated with age of introduction to solids.

## 3 RESULTS

### 3.1 Descriptive statistics

Eighty‐one mother–infant dyads were initially recruited. At 6 months, eight (10%) had withdrawn, and data from one dyad were removed because they had introduced solid food at the 3‐month telephone interview. This left a final sample of 72 mother–infant dyads. Eight mothers introduced solid food before infants were 17 weeks old, and six mothers followed WHO and DH guidelines and waited until infants were 6 months old before introducing solid food. The mean age infants were introduced to solid food was 20.57 weeks old (*SD* 3.13). When asked what type of solid food mothers fed their infant, 19.4% stated “only homemade,” 27.8% stated “mostly homemade,” 25% stated “about the same quantity of homemade and ready prepared food,” 15.3% stated “mostly ready prepared food,” and 12.5% stated “only ready prepared food.”

The sample had high breastfeeding rates, was affluent, well educated, and predominantly White British. Fifty‐three percent of infants (*n* = 38) were receiving breast milk at 6 months (71% of these had not introduced formula). Fifty‐six percent of families (*n* = 45) were in the highest income bracket (£351 or above per week). Forty‐six percent of mothers (*n* = 37) were degree educated. Fifty‐eight percent (*n* = 46) of mothers were White British, 12.5% Asian Pakistani (*n* = 10), and 10% White other (*n* = 8). Mean maternal body masss index at 6 months was 25.43 (*SD* 4.00). Eighteen percent of mothers (*n* = 13) scored 10 or greater on the EPDS and were therefore identified as showing symptoms indicative of possible depression.

### 3.2 Covariates

One‐tailed Pearson's correlations revealed that age of introduction to solid food was negatively associated with infant birth weight (*r* = −0.31, *p* = 0.004), and positively associated with maternal age (*r* = 0.20, *p* = 0.04), breastfeeding duration (*r* = 0.24, *p* = 0.02), and postnatal depression (*r* = 0.22, *p* = 0.03). These variables were therefore controlled in further analyses. Age of introduction to solid food was not associated with maternal BMI (*r* = 0.02, *p* = 0.42), education (*r* = 0.11, *p* = 0.19), or household income (*r* = 0.11, *p* = 0.19). There was no difference between male (*M* = 20.49, *SE* = 0.54) and female (*M* = 20.65, *SE* = 0.51) infants in the age at which they were introduced to solid food *t*(70) = −0.21, *p* = 0.83.

### 3.3 Age of introduction to solids and infant temperament

One‐tailed partial correlations were conducted to investigate relationships between age of introduction to solid food and infant temperament. Table 3 shows that age of introduction to solid food was negatively associated with infant smiling and laughter. Age of introduction to solid food was not related to other dimensions of infant temperament.

**Table 3:** Partial correlations (one‐tailed) between infant age introduced to solid food and temperament

		Activity level	Distress to limitations	Fear	Duration of orienting	Smiling & laughter	High intensity pleasure	Low intensity pleasure	Soothability	Falling reactivity	Cuddliness	Perceptual sensitivity	Sadness	Approach	Vocal reactivity
Age introduced to solids	*r*	0.07	−0.12	−0.03	−0.05	−0.30	−0.15	−0.13	−0.02	−0.10	−0.12	−0.03	0.05	−0.16	−0.11
	*p*	0.30	0.17	0.40	0.35	0.01	0.11	0.15	0.45	0.20	0.16	0.39	0.35	0.10	0.19
	*df*	66	66	66	66	66	66	66	66	66	66	66	66	66	66

*Note*. Covariates include: infant birth weight, maternal age, 6‐month Edinburgh Postnatal Depression Scale (EPDS), and breastfeeding duration.

### 3.4 Age of introduction to solids and maternal feeding and mealtime behaviours

One‐tailed partial correlations investigated relationships between age of introduction to solid food and maternal report of feeding behaviours during exclusive milk feeding at 3 months and observed maternal mealtime behaviours at 6 months. Table 4 shows that, after accounting for covariates, age of introduction to solid food was negatively associated with laissez‐faire milk feeding behaviours and positively associated with restrictive milk feeding behaviours. Age of introduction to solid food was not related to pressuring or responsive feeding behaviours during exclusive milk feeding. Table 4 also shows that, after accounting for covariates, age of introduction to solid food was positively related to observed maternal verbal involvement during a mealtime. Age of introduction to solid food was not associated with observed maternal controlling solid feeding behaviours (verbal or nonverbal), appropriateness, or sensitivity, as measured by the FIS.

**Table 4:** Partial correlations (one‐tailed) between age introduced to solid food and reported maternal feeding behaviours at 3 months and observed maternal mealtime behaviours at 6 months

		Age introduced to solids		
		*r*	*p*	*df*
3‐month reported milk feeding behaviours	Laissez‐faire	−0.28	0.01	66
	Pressure	0.09	0.23	66
	Restriction	0.21	0.04	66
	Responsive	−0.05	0.34	66
6‐month observed mealtime behaviours	Verbal involvement	0.23	0.05	48
	Verbal control	.19	.09	48
	Non‐verbal behaviour	0.15	0.15	48
	Appropriateness	0.12	0.20	48
	Sensitivity	0.13	0.18	48

*Note*. Covariates include: infant birth weight, maternal age, 6‐month Edinburgh Postnatal Depression Scale (EPDS), and breastfeeding duration.

### 3.5 Predictors of age of introduction to solid food

Hierarchical multiple regression analysis was performed to establish the significant predictors of age of introduction to solid food. Demographics significantly related to the age of introduction to solid food were entered in Step 1: infant birth weight, maternal age, breastfeeding duration, and EPDS (variables entered in Step 1 explained 21.2% of the variance in age of introduction to solid food). Predictors significantly related to the age of introduction to solid food were entered in Step 2: laissez‐faire and restrictive milk feeding behaviours, observed maternal verbal involvement, and the smiling and laughter dimension of the IBQR. Table 5 shows that the final model has two significant predictors of age of introduction to solid food, *R* ^2^ = 0.38; *F*(8, 46) = 3.57; *p* = 0.003, with lower birth weight and lower smiling and laughter being associated with later introduction to solid foods. After entry of the variables in Step 2, the total variance explained by the model was 38.3%, *R* ^2^ = 0.17; *F*(4, 46) = 3.18; *p* = 0.02.

**Table 5:** Hierarchical multiple regression predicting age of introduction to solid food

		*B*	*SE B*	β
	Constant	235.05	36.24	
Variables entered in Step 1	Infant birth weight	−0.02	0.01	−0.36**
	Maternal age	0.59	0.55	0.15
	Breastfeeding duration	0.07	0.05	0.25
	EPDS	−0.63	0.66	−0.12
Variables entered in Step 2	Laissez‐faire (IFSQ)	−2.43	2.31	−0.18
	Restrictive (IFSQ)	−0.29	2.71	−0.02
	Smiling and laughter (IBQ‐R)	−5.78	2.14	−0.35*
	Verbal involvement (FIS)	0.93	1.73	0.07

*Note*. EPDS: Edinburgh Postnatal Depression Scale; IFSQ: Infant Feeding Style Questionnaire; IBQ‐R: Infant Behaviour Questionnaire‐Revised; FIS: Feeding Interaction Scale.

* *p* = 0.01.

** *p* < 0.01.

The model predicts that as infant birth weight increases by 1 *SD* (336.70 g), the age at which they are introduced to solid food decreases by 0.36 *SD*. The SD for age of introduction to solid food is 22.00 days and so this constitutes a change of 7.92 days. Therefore, if infant birth weight increases by 336.70 g, the age at which they are introduced to solid food will decrease by 7.92 days.

As the IBQ‐R dimension of smiling and laughter increases by 1 *SD* (1.31 points), the age at which infants are introduced to solid food decreases by 0.35 *SD*. The SD for age of introduction to solid food is 22.00 days and so this constitutes a change of 7.7 days. Therefore, if smiling and laughter, as reported by mothers using the IBQ‐R, increases by 1.31 points, the age at which infants are introduced to solid food will decrease by 7.7 days.

## 4 DISCUSSION

The current study described relationships between infant temperament, maternal feeding behaviours, and the timing of introduction to solid food. The study also investigated whether demographic factors (infant birth weight, maternal age, breastfeeding duration, and EPDS score) and variables significantly related to age of introduction to solids (laissez‐faire and restrictive milk feeding behaviours, observed maternal verbal involvement, and the smiling and laughter dimension of the IBQR) predicted the timing of introduction to solid food. Infants were introduced to solid food at 5 months on average, with the earliest introduction at 13 weeks and the latest at 6.4 months. Infants introduced to solid food earlier were heavier at birth, had younger mothers who breastfed for shorter durations (or not at all), and scored lower for postnatal depression. Previous research has found mothers who introduce solid food earlier tend to be younger (Doub et al., 2015; Tatone‐Tokuda et al., 2009) and breastfeed for shorter durations (Wasser et al., 2011). Previous research has found an association between higher birth weight and earlier introduction to solid food, which is particularly strong if the mother had difficulty recognising her infant's hunger cues in the first 5 weeks postpartum (Kronborg, Foverskov, & Væth, 2014). Furthermore, Blissett and Farrow (2007) found that mothers of infants born heavier report using feeding styles characterised by more pressure at 1 year. Doub et al. (2015) and Wasser et al. (2011), however, did not find a relationship between age of introduction to solid food and infant birth weight. Unlike previous studies, we did not find significant relationships between age of introduction to solid food and household income, maternal education, or BMI (Doub et al., 2015; Tatone‐Tokuda et al., 2009; Wasser et al., 2011).

In preliminary analyses, we found that later introduction to solids was associated with higher postnatal depression scores. Previous research has not found a relationship between age of introduction to solid food and fewer symptoms of postnatal depression. In fact, Wasser et al. (2011) found that early complementary feeding was associated with more depressive symptoms. However, Gaffney, Kitsantas, Brito, and Swamidoss (2014) found the relationship between postnatal depression and timing of introduction to solids was not significant after controlling for covariates. Therefore, our findings were unexpected, and further studies investigating predictors of the age of introduction to solid food should consider inclusion of such a measure to examine the nature of this relationship in more depth. However, it is important to note that in the regression model, postnatal depression was not a significant predictor of age of introduction to solid food, demonstrating that infant characteristics were more important predictors of timing.

After controlling for covariates, analyses revealed mothers who rated their 6‐month‐old infants as higher in smiling and laughter introduced solid food earlier. This is contradictory to the hypothesis that infants perceived to be more physically active, who show more distress and sadness and who are more difficult to soothe, would be introduced to solid food earlier, and previous research, which found that infants rated higher in distress and activity level, was introduced to solid food earlier (Wasser et al., 2011). However, the lack of an association between age of introduction to solid food and infant distress and activity level is consistent with other research, which did not find a relationship between difficult infant temperament and early introduction to solid food (Doub et al., 2015; Tatone‐Tokuda et al., 2009). There were no other relationships between maternal report of infant temperament and age of introduction to solid food, which suggests that other aspects of temperament do not appear to be key correlates of age of introduction to solid food.

Mothers who scored highly on laissez‐faire feeding behaviours were those who did not keep track of how much milk their infant drank at 3 months, which in turn was associated with earlier introduction of solid food. Mothers who reported keeping track of how much milk their infant drank at 3 months may have been more anxious about their infant's feeding and may have been keener to stick to current guidelines regarding when to introduce solid food. Arden (2010) found an association between later introduction to solids and a focus on the current recommendation (to introduce solids at 6 months) as important. Previous research has also suggested that mothers may use more controlling feeding practices during infancy if they would like to monitor milk intake, offer feeds at certain times, or if they are anxious about their infant's weight or other health difficulties (Brown & Lee, 2013; Grøvslien & Grønn, 2009).

The current study also found that mothers who introduced solid food earlier exhibited lower verbal involvement during an observed mealtime. Low scores in verbal involvement indicate mothers who initiated less (or no) conversation or spontaneous comments during observed mealtimes. It is possible that less parent–child interaction during feeding may put parents at risk of missing their infant's communications regarding food and the mealtime. Kronborg et al. (2014) found that mothers who reported not recognising early infant cues of hunger introduced their infants to solid food earlier. We did not, however, find an association between responsive milk feeding behaviours and the timing of introduction to solid food. This is contrary to findings of Doub et al. (2015), who found that mothers who reported less responsiveness to their infant's hunger and satiety cues (using the IFSQ) introduced solid food earlier.

The current study also failed to find that mothers who used more pressure introduced solid food earlier. This suggests that maternal pressure of infants to drink more milk is not linearly related to the timing of introduction to solid food. The lack of a relationship in the current study between pressuring feeding behaviours and age of introduction to solid food might be because mothers were not concerned with introducing solid food in order to help their infants gain weight. Instead, we found that mothers who, whilst exclusively milk feeding, reported more restrictive feeding behaviours introduced solid food later. Therefore, it may be that mothers who were more concerned about their infants maintaining a healthy weight adhered to current guidelines regarding when to introduce solid foods. As previously noted, mothers who believe current recommendations to be important have been found to introduce their infants to solid food later (Arden, 2010).

Earlier introduction to solid food was best predicted by heavier infant birth weight and higher IBQ‐R ratings for smiling and laughter (the degree to which their mothers perceive them to smile and laugh during play or general caretaking activities) over and above maternal age, breastfeeding duration, postnatal depression, reported feeding behaviours during the period of exclusive milk feeding, and verbal involvement during an observed meal. It is possible that heavier infants have larger appetites and make greater demands or are perceived to be more demanding and hungry by their parents. Parents may therefore introduce solid food earlier to meet their infant's (perceived) demands. This is likely, given that previous research has shown that rapid weight gain in the first 6 weeks and parents' perception that their infant was hungry were two of the strongest independent predictors of earlier age at weaning (Wright, Parkinson, & Drewett, 2004). This is also consistent with the finding that the most common reasons mothers introduce solid food are perceptions that their baby is hungry, unsettled, and not getting enough sleep (Brown & Rowan, 2016). The perception that heavier infants may need solid food earlier, however, is erroneous, as most foods used early on in weaning are low in calories. Research has shown that total energy intake and weight gain do not differ between breastfed infants given solids before 6 months and infants breastfed exclusively until 6 months (Heinig, Nommsen, Peerson, Lonnerdal, & Dewey, 1993; Smith & Becker, 2016).

Furthermore, infants who smile and laugh more during play after accomplishing tasks, and during bathing, washing, and dressing may be perceived as happier and more sociable by their mothers. These infants are also likely to communicate similar interest and enthusiasm during other activities, such as in feeding and mealtime situations. It is therefore possible that mothers may perceive this positive communication during mealtimes as interest in food, and so introduce solid food earlier. Future research should seek to explore this further, as it is possible that parents may be misinterpreting more general interest and engagement in the social environment as signs of readiness for solid food (Brown & Rowan, 2016). Although this was an exploratory study and replication of findings is required, information of this kind may be useful to health professionals and could inform guidance given to parents regarding the introduction of solid food. For example, if an infant is sociable, high in smiling and laughter, parents can be reassured that this interest may not be specific to food and eating and can be encouraged to interact with their child in a variety of ways other than introducing solid food earlier than is recommended.

There is an interesting complexity in the findings of the study. Our correlation analysis showed that infants introduced to solid food earlier were rated by their mothers as showing more smiling and laughter, yet the mothers who introduced solid food earlier were more laissez‐faire and showed less restriction in milk‐feeding behaviours, as well as being less likely to demonstrate verbal involvement within observed interactions. However, in the regression model, only infant characteristics were significant predictors of the timing of introduction to solid food. Nonetheless, the evolution of the interaction between maternal engagement and infant temperament across time, and its role in eliciting introduction to solid food, requires further longitudinal research.

It is important to consider the strengths and potential limitations of this work. Parental feeding practices are responsive to the child; parents take individual characteristics and eating behaviours of their children into account and adapt their feeding practices accordingly (Shloim, Edelson, Martin, & Hetherington, 2015). It is therefore possible that an infant's response to solid food may shape the feeding behaviours exhibited by their parents. The current study assessed maternal feeding behaviours before and after the introduction of solid food, so it was possible to explore relationships between the timing of introduction to solid food and maternal feeding behaviours during both the period of exclusive milk feeding and after the introduction of solids. However, maternal feeding behaviours at 3 and 6 months were assessed differently. Observations of mealtimes at 6 months allowed for the collection of objective information regarding feeding behaviours exhibited by mothers when feeding solid food. Feeding behaviours at 3 months, on the other hand, were assessed indirectly via maternal self report. Although the study adopted a longitudinal design and filmed interactions between mothers and infants, the sample size was small for questionnaire‐based data.

The current study did not record the weaning style adopted by parents. It is therefore not possible to investigate how many mothers used a baby‐led weaning style versus more traditional styles of offering pureed foods or how weaning style is related to infant temperament and maternal milk feeding behaviours assessed by the IFSQ. In addition, previous research has found maternal personality and anxiety are associated with breastfeeding (Brown, 2014) and the timing and method of introduction to solid food (Brown, 2016). These factors were not assessed in the current study. Future work should consider these factors, particularly given their association with infant temperament. Lastly, the sample were predominantly White (58% White British) with a higher level of education compared with the national average (Statistics., 2011), and this homogeneity may explain why we did not see significant demographic effects. Future work should explore a wider range of demographic, socio‐economic, and psychosocial factors with regard to the timing and method of introduction to solid food.

## 5 CONCLUSIONS

Infant characteristics, namely, their birth weight and the extent to which they are perceived to smile and laugh during play and caretaking activities, seem to be key predictors of when they will be introduced to solid food. These characteristics seem to be more important than maternal age, breastfeeding duration, postnatal depression, and feeding behaviours. Information of this kind is important, given the lack of adherence to current guidelines in the United Kingdom, and the fact that signs of readiness for solid food are commonly misinterpreted by parents (Brown & Rowan, 2016). Further work is therefore required to investigate infant characteristics that affect parental perception of readiness for the introduction of solid food.

## CONFLICTS OF INTEREST

The authors declare that they have no conflicts of interest.

## CONTRIBUTIONS

SLR conceptualised and designed the study, collected, analysed and interpreted the data, wrote the initial manuscript, and approved the final manuscript as submitted. JB conceptualised and designed the study, supervised data collection, contributed to the interpretation of data, critically reviewed the manuscript, and approved the final manuscript as submitted.
